# Is Milk Thistle a Cardioprotective Ally or a Thrombotic Threat?

**DOI:** 10.1016/j.jaccas.2025.104999

**Published:** 2025-09-17

**Authors:** Eva Lindner, Musa'ab Moh'd Alhmouz, Raghad Abuhalimeh, Lina Abuhalimeh, Jason Chin, Mohammad Qadura, Houssam Younes, Batool Jamal Abuhalimeh

**Affiliations:** a Heart, Vascular & Thoracic Institute, Cleveland Clinic Abu Dhabi, Abu Dhabi, UAE; b University of Jordan, Amman, Jordan

**Keywords:** deep vein thrombosis (DVT), milk thistle, silibinin

## Abstract

**Background:**

The supplement milk thistle has been known to have estrogen-mimicking effects, raising the concern for a possible factor contributing to venous thrombosis.

**Case Summary:**

We present a case of a 41-year-old male of Caucasian descent who presented to our clinic with a deep vein thrombosis with no known provoking factors or underlying hypercoagulable etiologies, occurring in the context of milk thistle use. Thorough workup was performed, and all results were negative. Following a multidisciplinary discussion and literature reviews, it was determined that the patient's deep vein thrombosis was possibly associated with the use of milk thistle.

**Discussion:**

Understanding the provoking factors contributing to venous thrombosis is essential for effective management and prevention of recurrence. We provide a comprehensive review of the latest evidence on milk thistle's potential association with thrombosis and its roles in cardiovascular health.

**Take-Home Message:**

Milk thistle's complex cardiovascular effects and estrogenic activity highlight the need for multidisciplinary care and further clinical trials.

## Graphical Abstract

**Figure:**
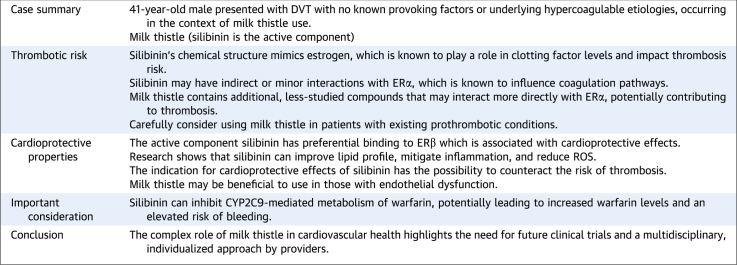

## Background

Venous thromboembolic disease poses a significant clinical burden with substantial morbidity and mortality. With the increasing prevalence of venous thromboembolism, now affecting approximately 10 million people annually worldwide, understanding the provoking factors contributing to venous thrombosis is essential for effective management and prevention of recurrence.^1^ Milk thistle (*Silybum marianum*) is considered a safe supplement and is used for liver support due to its active components, which have antioxidant and anti-inflammatory properties. Its active component, silibinin, however, has been shown to interact with estrogen receptors (ERs), possibly mimicking estrogen's effects. Because estrogen is known to influence coagulation pathways, the use of milk thistle may increase the possibility of thrombosis.

**Take-Home Messages**

• Milk thistle has complex cardiovascular effects; hence, a multidisciplinary approach is essential to assess supplement use in patients with thrombotic events, ensuring a balanced evaluation of risks and benefits in this patient population.
• The role of milk thistle in cardiovascular health and thrombosis risk remains debatable given its estrogenic activity, emphasizing the need for future clinical trials to verify the function and the use of milk thistle.

## History of presentation

A 41-year-old gentleman of Caucasian descent with no significant past medical history presented to the vascular medicine clinic with the complaint of left-lower-extremity swelling and pain that started 24 hours prior to presentation.

## Past medical history

The patient denied personal or family history of hypercoagulable disease. He also denied recent history of prolonged immobilization, travel, flu-like symptoms, sick contacts, positive COVID testing, and injuries or trauma prior to presentation.

## Differential diagnosis

Baker's cyst, popliteal artery aneurysm, muscle strain, knee joint pathology, and lower-extremity cellulitis.

## Investigation

A duplex ultrasound was performed which revealed a deep vein thrombosis (DVT) in the posterior tibial and popliteal veins of the left lower extremity. The patient was subsequently started on rivaroxaban (Bayer) for treatment of DVT. Meanwhile, hypercoagulable workup was found normal, including lupus anticoagulant, anticardiolipin antibodies, beta-2 glycoprotein, protein S, protein C, antithrombins, prothrombin, homocysteine, and factor V. In addition, age-appropriate malignancy screening was found normal. On further questioning, the patient states that 7 months prior, he started using supplements purchased off the counter, including vitamin D, milk thistle, and vitamin B complex.

## Management

The patient was treated with rivaroxaban for a total of 6 months, during which he reported compliance and tolerance of anticoagulation therapy. Following a multidisciplinary discussion, it was determined that the patient's DVT was possibly associated with the use of milk thistle.

## Outcome

After discontinuing milk thistle, the patient returned for follow-up at the vascular clinic 1 year after the initial diagnosis, with no recurrent DVTs and in good health.

## Discussion

Milk thistle (*S. marianum*), a widely used herbal supplement, is known for its hepatoprotective, antioxidant, and anti-inflammatory properties, which are primarily attributed to its active flavonolignan complex, silymarin.^2^^,^^3^ Among its components, silibinin has been identified as a bioactive compound with potential estrogenic activity through its interaction with ERs.^3^ Given the well-established role of estrogen in coagulation pathways, concerns have emerged regarding the potential prothrombotic effects of milk thistle, particularly in individuals with predisposing risk factors for thrombosis.^4^ Contradictory data in the literature regarding the relationship between milk thistle and venous thrombosis highlight the need for further research to assess the safety of this supplement, particularly in individuals at substantial risk of thrombotic events. In the context of this case, we hereby evaluate both perspectives on this issue and a review on the existing evidence, highlighting the need for increased awareness and further investigation.

## Mechanisms of Estrogenic Activity and Thrombosis Risk

Estrogen affects coagulation by elevating clotting factor levels and decreasing anticoagulant protein activity, thereby increasing the risk of thrombosis.^4^ Silibinin is known to activate both ERs, ERα and ERβ, which regulate cardiovascular, thrombosis, and inflammatory pathways.^2^, ^3^, ^4^, ^5^ By modulating ER activity, particularly ERβ, silibinin exerts antioxidative and endothelial-stabilizing properties that contribute to its cardioprotective effects. While silibinin has shown cardiovascular benefits, such as improved endothelial function and reduced oxidative stress, its estrogenic activity raises concerns about a potential prothrombotic effect.^6^, ^7^, ^8^

Preclinical studies suggest that silibinin exhibits selective estrogen receptor modulator-like activity, with a higher affinity for ERβ.^9^^,^^10^ Activation of ERβ is associated with anti-inflammatory and cardioprotective effects, while ERα activation has been linked to prothrombotic mechanisms.^4^^,^^5^ Despite its preferential binding to ERβ, the potential interaction of silibinin with ERα raises concerns about whether it could contribute to an increased thrombosis risk, especially in individuals with pre-existing prothrombotic conditions.

Moreover, while silibinin is the primary active component of milk thistle, the supplement also contains numerous other compounds, the effects of which are less well understood.^9^ The limited research on these isomeric forms, including their chemistry and biological behavior, raises the possibility that other components in the mixture may contribute to potential risk of thrombosis. Therefore, the variability in data could stem from interactions between these lesser-studied compounds and individual physiological differences.

In this case, after discontinuing milk thistle but continuing anticoagulation therapy, the patient's symptoms resolved and did not reoccur. While the data suggest a potential link between milk thistle, its estrogen-like effects, and an increased risk of thrombosis, its stronger affinity for ERβ and known cardiovascular benefits make it difficult to definitively establish milk thistle's role in the patient's thrombotic event.

## Evidence on Milk Thistle and Endothelial-Stabilizing Properties

Despite the possible link between milk thistle and thrombosis, direct clinical evidence remains limited. Some studies suggest that silibinin may support cardiovascular health through its antioxidant properties and ability to stabilize the endothelium.^6^, ^7^, ^8^ Although ERα activation has been linked to prothrombotic effects in preclinical research, silibinin's stronger affinity for ERβ—associated with anti-inflammatory and cardioprotective actions—indicates a potentially protective cardiovascular profile.^6^, ^7^, ^8^, ^9^, ^10^ Furthermore, the supplement's antioxidative and anti-inflammatory effects may counteract any minor thrombotic risks. Research has indicated that silibinin improves lipid profiles, enhances endothelial nitric oxide synthase expression, and reduces oxidative stress markers, such as reactive oxygen species. In addition, it mitigates inflammation by lowering levels of adhesion molecules, endothelin-1, and angiotensin-II, mechanisms closely resembling those of estrogen therapy. Silibinin has also been found to inhibit low-density lipoprotein as well as oxidation and monocyte adhesion, key factors in atherosclerosis and thrombus formation.^6^, ^7^, ^8^ These beneficial antioxidant properties suggest a more protective role against vascular damage, reducing the risk of thrombotic events rather than exacerbating them. Thus, silibinin may be considered a viable supplement in cardiovascular health management, particularly for individuals with endothelial dysfunction.

The use of milk thistle in patients on anticoagulant therapy requires careful consideration. Silibinin can interact with CYP2C9, an enzyme critical for the metabolism of warfarin. Studies have shown that silibinin can inhibit CYP2C9-mediated metabolism of warfarin, potentially leading to increased warfarin levels and an elevated risk of bleeding. This interaction was observed in a clinical case where a patient's international normalized ratio increased significantly after starting a milk thistle supplement but normalized upon discontinuation of the supplement.^11^^,^^12^

## Conclusions

The debate surrounding milk thistle's role in cardiovascular health and thrombosis risk highlights the complexity of its estrogenic activity. While evidence suggests significant cardioprotective and antioxidative benefits, concerns regarding its potential prothrombotic effects require further investigation. Future clinical trials should aim to clarify these risks, particularly in populations predisposed to thrombosis or those on anticoagulant therapy. Until then, healthcare providers must weigh the potential benefits and risks of the use of milk thistle on a case-by-case basis, ensuring patient safety remains the priority.

**Visual Summary:** Summary of the Cardiovascular Effects of Milk Thistle

Case summary	41-year-old male presented with DVT with no known provoking factors or underlying hypercoagulable etiologies, occurring in the context of milk thistle use. Milk thistle (silibinin is the active component)
Thrombotic risk	Silibinin's chemical structure mimics estrogen, which is known to play a role in clotting factor levels and impact thrombosis risk. Silibinin may have indirect or minor interactions with ERα, which is known to influence coagulation pathways. Milk thistle contains additional, less-studied compounds that may interact more directly with ERα, potentially contributing to thrombosis. Carefully consider using milk thistle in patients with existing prothrombotic conditions.
Cardioprotective properties	The active component silibinin has preferential binding to ERβ which is associated with cardioprotective effects. Research shows that silibinin can improve lipid profile, mitigate inflammation, and reduce ROS. The indication for cardioprotective effects of silibinin has the possibility to counteract the risk of thrombosis. Milk thistle may be beneficial to use in those with endothelial dysfunction.
Important consideration	Silibinin can inhibit CYP2C9-mediated metabolism of warfarin, potentially leading to increased warfarin levels and an elevated risk of bleeding.
Conclusion	The complex role of milk thistle in cardiovascular health highlights the need for future clinical trials and a multidisciplinary, individualized approach by providers.

DVT = deep vein thrombosis; ERα = estrogen receptor α; ERβ = estrogen receptor β; ROS = reactive oxygen species.

## Funding Support and Author Disclosures

The authors have reported that they have no relationships relevant to the contents of this paper to disclose.
